# Long-term survival in patients with septic acute kidney injury is strongly influenced by renal recovery

**DOI:** 10.1371/journal.pone.0198269

**Published:** 2018-06-05

**Authors:** Marco Fiorentino, Fadi A. Tohme, Shu Wang, Raghavan Murugan, Derek C. Angus, John A. Kellum

**Affiliations:** 1 The Center for Critical Care Nephrology, Department of Critical Care Medicine, University of Pittsburgh, Pittsburgh, PA, United States of America; 2 Department of Emergency and Organ Transplantation, Nephrology, Dialysis and Transplantation Unit, University of Bari, Bari, Italy; 3 The CRISMA Center, Department of Critical Care Medicine, University of Pittsburgh, Pittsburgh, PA, United States of America; 4 Renal & Electrolyte division, Department of Medicine, University of Pittsburgh, Pittsburgh, PA, United States of America; 5 Graduate School of Public Health, University of Pittsburgh, Pittsburgh, PA, United States of America; Bambino Gesù Children's Hospital, ITALY

## Abstract

**Background:**

Several studies have shown that long-term survival after acute kidney injury (AKI) is reduced even if there is clinical recovery. However, we recently reported that in septic shock patients those that recover from AKI have survival similar to patients without AKI. Here, we studied a cohort with less severe sepsis to examine the effects of AKI on longer-term survival as a function of recovery by discharge.

**Methods:**

We analyzed patients with community-acquired pneumonia from the Genetic and Inflammatory Markers of Sepsis (GenIMS) cohort. We included patients who developed AKI (KDIGO stages 2–3) and defined renal recovery as alive at hospital discharge with return of SCr to within 150% of baseline without dialysis. Our primary outcome was survival up to 3 years analyzed using Gray’s model.

**Results:**

Of the 1742 patients who survived to hospital discharge, stage 2–3 AKI occurred in 262 (15%), of which 111 (42.4%) recovered. Compared to recovered patients, patients without recovery were older (75 ±14 vs 69 ±15 years, p<0.001) and were more likely to have at least stage 1 AKI on day 1 (83% vs 52%, p<0.001). Overall, 445 patients (25.5%) died during follow-up, 23.4% (347/1480) for no AKI, 28% (31/111) for AKI with recovery and 44.3% (67/151) for AKI without recovery. Patients who did not recover had worse survival compared to no AKI (HR range 1.05–2.46, p = 0.01), while recovering patients had similar survival compared to no AKI (HR 1.01, 95%CI 0.69–1.47, p = 0.96). Absence of AKI on day 1, no in-hospital renal replacement therapy (RRT), higher Apache III score and higher baseline SCr were associated with recovery after AKI.

**Conclusions:**

In patients with sepsis, recovery by hospital discharge is associated with long-term survival similar to patients without AKI.

## Introduction

Controversy exists as to whether patients who develop acute kidney injury (AKI) but fully recover renal function have decreased long-term survival and increased risk for end-stage renal disease (ESRD) compared to patients who do not develop AKI. For example, studies of patients undergoing major surgery suggest that AKI among patients with no baseline chronic kidney disease (CKD), even with recovery of renal function, is associated with an increased risk of death and ESRD in subsequent years [1–3]. Similarly, Linder and colleagues found that critically ill patients with one episode of mild AKI (stage 1) have significantly lower 10-year survival rates compared to patients with no AKI [4]. However, these studies did not specifically examine septic AKI. Sepsis is the leading cause of AKI among critically ill patients [5, 6], and septic AKI is associated with a higher risk of death and increased health-resource utilization as compared to other causes of AKI [7], even in patients without severe sepsis [8]. Importantly, we recently found that approximately half of all patients who develop AKI in the setting of septic shock completely recover renal function by hospital discharge, and these patients appear to have similar 1-year survival rates to patients without AKI [9]. Conversely, patients without recovery have very poor outcomes. Therefore, it is unclear whether sepsis induces a unique AKI phenotype and whether the consequences of recovery are different and more significant even in patients without severe sepsis. If so, this would be important because most cases of septic AKI are already seen at presentation [9] and thus, prevention is not feasible.

Furthermore, identifying factors that can predict recovery in septic AKI could help define subgroups of individuals that are more likely to benefit from interventions [10]. Finally, understanding the effects of renal recovery at hospital discharge on long-term outcomes after AKI can help inform clinicians about prognosis and may have important implications for follow-up of these patients. Thus, our primary goal was to determine the relationship between renal recovery and long-term survival in a cohort of patients with AKI secondary to community-acquired pneumonia, a leading infectious cause of hospitalization in developed countries. Our secondary goal was to identify factors associated with non-recovery of renal function.

## Materials and methods

### Study population

We used the Genetic and Inflammatory Markers of Sepsis (GenIMS) study. Details of this study have been published previously [11]. Briefly, GenIMS was a prospective inception cohort of adult patients hospitalized with community-acquired pneumonia who presented to 28 teaching and non-teaching hospitals in the United States between 2001 and 2003. Eligible patients were ≥18 years old and had a clinical and radiological diagnosis of pneumonia using criteria by Fine *et al* [12]. The institutional review board of the University of Pittsburgh approved the study, and we obtained written informed consent from all participants or their proxies. We limited our primary analysis to hospital survivors since the current analysis focuses on long-term survival. The results of this study are reported with adherence with the Strengthening Reporting Observational studies in Epidemiology (STROBE) guidelines.

### Definition of AKI and determination of baseline renal function

We defined AKI as stages 2–3 AKI using the Kidney Disease Improving Global Outcomes (KDIGO) classification system [13]. KDIGO stage 2 AKI is defined as a 2.0–2.9 times baseline increase in serum creatinine (SCr). KDIGO stage 3 AKI is defined as an increase in SCr to ≥3.0 times baseline or an increase in SCr to ≥4 mg/dL or initiation of renal replacement therapy [13]. Stages were determined based on SCr and urine output (UO) data. For patients with premorbid SCr, the most recent value in the last 12 months prior to hospitalization was used as baseline SCr. For patients with no known premorbid SCr and no known medical history of chronic kidney disease, premorbid SCr was estimated using the Modification of Diet in Renal Disease (MDRD) equation, as recommended by the Acute Disease Quality Initiative [14]. The lower SCr value from either hospital admission creatinine or the MDRD creatinine was then selected as the baseline value.

### Definition of renal recovery

Renal recovery status was determined at hospital discharge. Recovery was defined according to international consensus criteria [15] and prior literature [16, 17] as a return of SCr to within 150% of baseline or less without need for renal replacement therapy. Non-recovery was defined as need for renal replacement therapy (RRT) within 48 hours from discharge or no return of SCr to ≤150% of baseline.

### Study outcomes

Our primary outcome was survival at a maximum follow-up of 3 years from the date of hospitalization (maximum follow-up available from the parent trial). Secondary outcomes included incident ESRD after hospital discharge and variables associated with recovery. Study coordinators ascertained deaths in hospital, and post-discharge mortality was ascertained by telephone and National Death Index search. Incident ESRD, defined as incident dialysis or kidney transplantation, was determined over the first year after hospitalization through query of the United States Renal Data System (USRDS).

### Statistical analysis

For baseline characteristics, either Pearson’s chi-squared test or Fisher’s exact test was used to compare categorical variables between patients without AKI, those with AKI who recovered, and those with AKI but without recovery. Continuous variables were compared using Kruskal-Wallis rank tests or ANOVA as non-parametric and parametric methods, respectively. First, Kaplan-Meier curves were constructed to determine unadjusted survival rates for the different subgroups of renal recovery. Peto-Peto-Prentice test was used to determine statistical significance between these subgroups. Second, we used Schoenfeld residual plots to test the proportional hazards assumption for continuous variables and log-log plot for categorical variables. Renal recovery was found to violate the proportional hazards assumption, so we built a multivariable Gray’s model with backward selection of variables (using p-value <0.2 in univariable Gray’s model as a cutoff for inclusion). The final Gray’s model was adjusted for age, sex, Charlson comorbidity index and day 1 pneumonia severity index (PSI) without age (with non-time varying coefficients). Hazard ratios (HR) of non-recovery vs no AKI from the Gray’s model are reported as the minimum to maximum covariate effect during 10 time intervals and p-values are reported for the overall model. Third, because commonly used general severity of illness scores incorporate either SCr or blood urea nitrogen, we used the non-renal domains of the sequential organ failure assessment (SOFA) score on day 1 to estimate severity of illness independent of AKI status. Because few patients reached the outcome of ESRD over the first year after hospital discharge, we did not attempt to build a model for ESRD. In addition, we built a multivariable logistic regression model with stepwise selection of variables (using a p value <0.2 as a cutoff for inclusion in the final model) to determine which factors are associated with recovery of renal function after AKI. This analysis was limited to those patients who developed AKI and predictors were present at baseline or on day 1 only. All patients who developed AKI and died prior to discharge were considered non-recovered. The variable “chronic kidney disease (CKD) at baseline” was inserted into the model due to its clinical significance. In our data set, cardiac disease and AKI on day 1 don’t have complete data. We dropped those subjects who had missing data in model fitting and then used MICE (Multivariate Imputation by Chained Equation) to conduct imputation as a sensitivity analysis [18]. A receiver operating characteristic (ROC) curve was constructed using factors that remained in the final model. Predictors of recovery were reexamined in sensitivity analysis, including all patients with AKI, not only those who survived to hospital discharge. P values <0.05 were considered to be statistically significant. All analyses were conducted using Stata 14.1 (College Station, TX), SAS 9.4 (Cary, NC) and R 14.0 (URL: https://www.r-project.org/).

## Results

### Baseline characteristics and AKI status

**Fig 1:**
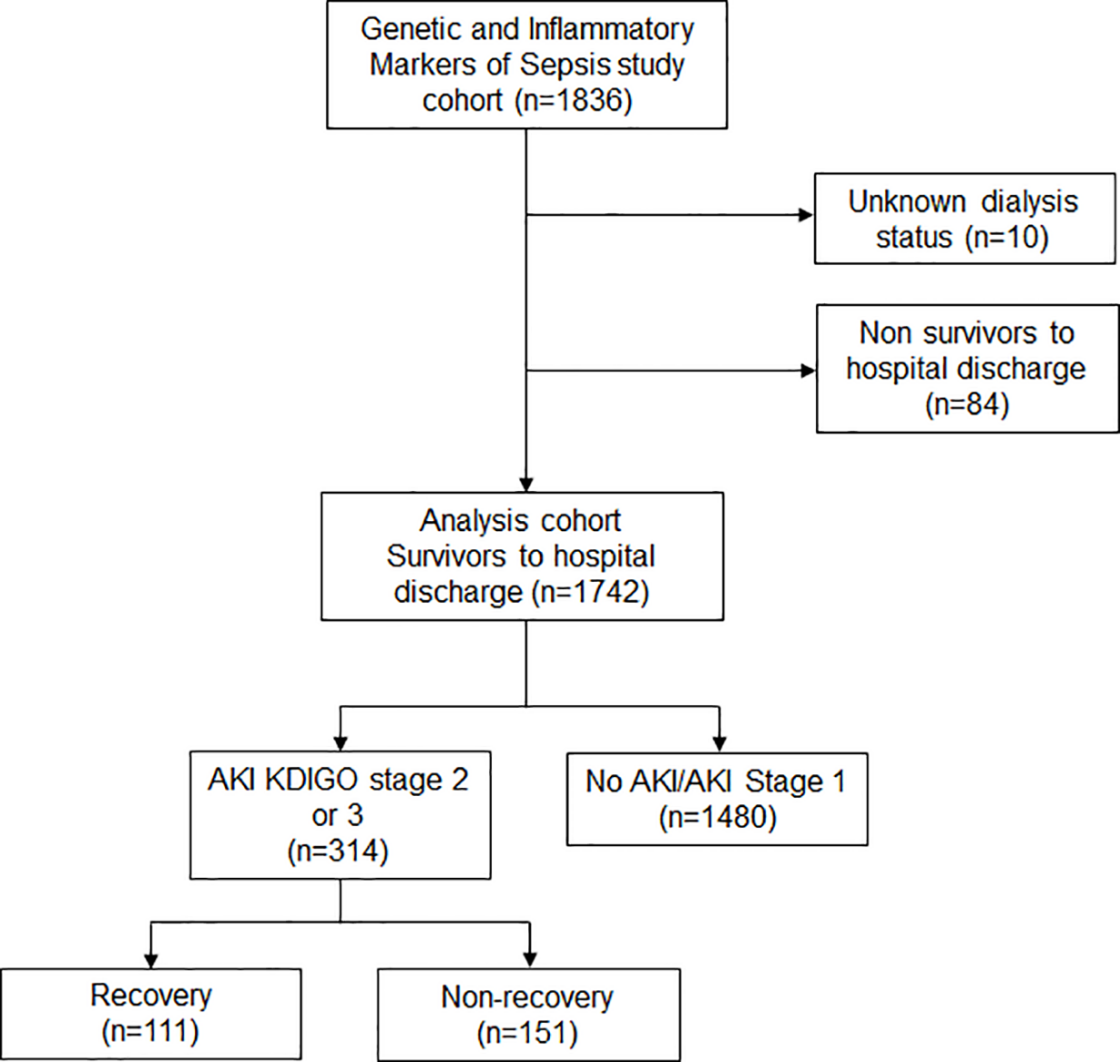
Flow chart of study population.

A total of 1836 patients were enrolled in the GenIMS study, of which 1752 survived to hospital discharge. We excluded 10 patients with unknown dialysis status at 1 year (**Fig 1**). In our final study cohort (n = 1742), 262 (15%) developed stage 2–3 AKI, of which 111 (42.4%) had recovery of renal function by hospital discharge. Baseline characteristics and hospital outcomes are shown in **Table 1**. Results are stratified by AKI and by recovery. Compared to patients with recovery and those without AKI, patients without recovery were older (76 ± 14 vs 69 ± 15 for patients with recovery vs 67 ± 17 for no AKI). Patients without recovery were more likely to have AKI on day 1 compared to patients with recovery (83% vs 52%). Patients not recovering had lower severity of AKI (43% had severe AKI (stage 3) vs 66% for those recovering), lower baseline serum creatinine (0.90 ± 0.15 vs 1.01 ± 0.64) and had a shorter median hospital length of stay (7 [interquartile range, IQR 5–11] days vs 11 [IQR 7–17] days) compared to those recovering (**Table 1**).

**Table 1:** Baseline characteristics and hospital outcomes of the study population.

	All patients (n = 1742)	No AKI (n = 1480)	AKI with Recovery (n = 111)	AKI without Recovery (n = 151)
Age, years, mean (SD)	68 (17)	67 (17)	69 (15)	75 (14)
Male sex	901 (52%)	768 (52%)	60 (54%)	73 (48%)
White race	1411 (81%)	1193 (81%)	86 (77%)	132 (87%)
CCI, mean (SD)	1.8 (2.2)	1.8 (2.2)	1.7 (2.0)	2.2 (2.3)
Chronic kidney disease	32 (2%)	14 (1%)	6 (5%)	12 (8%)
Cardiac disease*	441 (25%)	360 (24%)	27 (30%)	54 (36%)
Lung disease	675 (39%)	586 (40%)	42 (38%)	47 (31%)
Diabetes mellitus	339 (19%)	274 (19%)	25 (23%)	40 (26%)
Baseline SCr, mg/dL, mean (SD)	0.90 (0.25)	0.89 (0.20)	1.01 (0.64)	0.90 (0.15)
Baseline eGFR, mean (SD)	85 (21)	86 (22)	78 (17)	78 (10)
APACHE III score on day 1, mean (SD)	39 (13)	38 (12)	50 (14)	46 (14)
Non-renal daily SOFA score, mean (SD)	1.7 (1.4)	1.6 (1.3)	2.5 (2.1)	2.1 (1.6)
Pneumonia Severity Index (PSI) on day 1, mean (SD)	97 (36)	93 (34)	118 (37)	123 (31)
AKI on day 1*	354 (20%)	173 (12%)	56 (52%)	125 (83%)
Severity of AKI				
No AKI	1189 (68%)	1189 (80%)	0 (0%)	0 (0%)
Stage 1	291 (18%)	291 (20%)	0 (0%)	0 (0%)
Stage 2	124 (7%)	0 (0%)	38 (34%)	86 (57%)
Stage 3	138 (8%)	0 (0%)	73 (66%)	65 (43%)
Required ICU admission	233 (13%)	109 (7%)	79 (71%)	45 (30%)
Hospital length of stay in days, median (IQR)	7 (4–8)	5 (4–8)	11 (7–17)	7 (5–11)
Developed severe sepsis	483 (28%)	329 (22%)	75 (68%)	79 (52%)
In-hospital RRT	15 (1%)	0 (0%)	3 (3%)	12 (8%)

* Not all patients had information about baseline cardiac disease and presence of AKI on day 1

AKI: acute kidney injury; CCI: Charlson comorbidity index; SCr: serum creatinine; eGFR: estimated glomerular filtration rate; APACHE III: acute physiologic and chronic health evaluation III; SOFA: sequential organ failure assessment; ICU: intensive care unit; RRT: renal replacement therapy

### Post-hospitalization deaths and incident ESRD

Out of a total of 445 deaths, 113 (25.4%) were recorded at a maximal follow-up time of 3 years. For patients without AKI 347/1480 (23.4%) died during follow-up, while 31/111 (28%) of patients with AKI but who recovered initially, died and 67/151 (44.3%) died among those who did not recover by hospital discharge (p<0.0001). Median follow-up time was 1.7 years (IQR 1.2–2.1 years). Only 11 patients (0.63%) developed incident ESRD over the first year after hospitalization (maximum follow-up for this endpoint). Patients with incident ESRD had a mean age of 66 ± 13 years, 45% were of non-white race, 27% had a known history of CKD at baseline, and their average baseline eGFR was 64 ± 24 mL/min/1.73m^2^. 91% of patients who developed ESRD had severe AKI (KDIGO stage 3), 80% did not recover by hospital discharge, 73% had severe sepsis and 55% received RRT during hospitalization (**Table 2**).

**Table 2:** Post-hospitalization ESRD.

	No ESRD (n = 1731)	ESRD (n = 11)
Age, years, mean (SD)	68 (17)	66 (13)
Non-white race	326 (19%)	5 (45%)
Chronic kidney disease	29 (1.7%)	3 (27%)
Baseline eGFR, mean (SD)	85 (21)	64 (24)
Non-renal daily SOFA score, mean (SD)	1.7 (1.4)	2.1 (2.1)
AKI	252 (31%)	10 (91%)
Severe	128 (51%)	10 (100%)
Non-recovery	143 (57%)	8 (80%)
Developed severe sepsis	475 (27%)	8 (73%)
In-hospital RRT	9 (0.5%)	6 (55%)

ESRD: end-stage renal disease; eGFR: estimated glomerular filtration rate; SOFA: sequential organ failure assessment; RRT: renal replacement therapy

### Survival models

**Fig 2:**
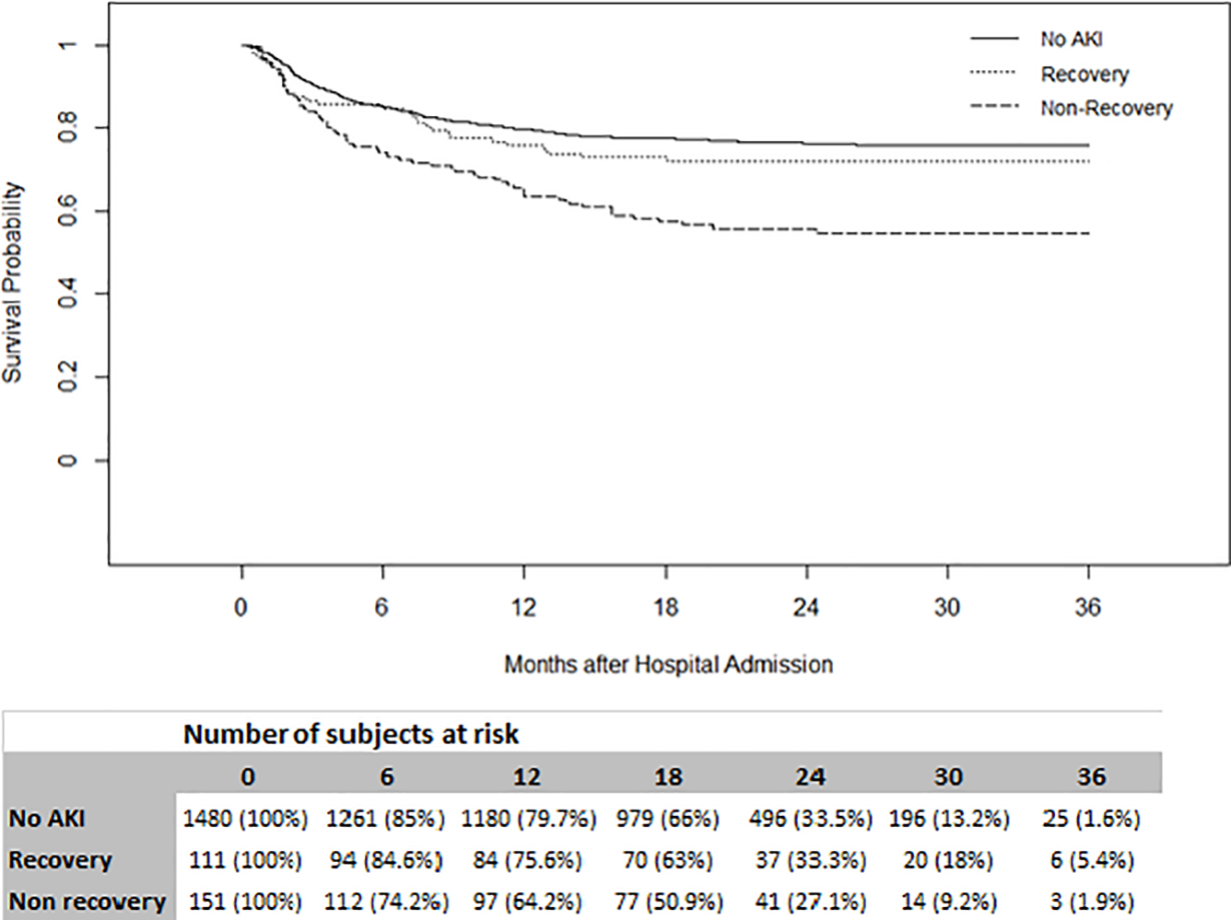
Kaplan-Meier survival curves stratified by recovery status. The three groups are significantly different overall, p < 0.001 (Peto-Peto-Prentice test).

**Fig 3:**
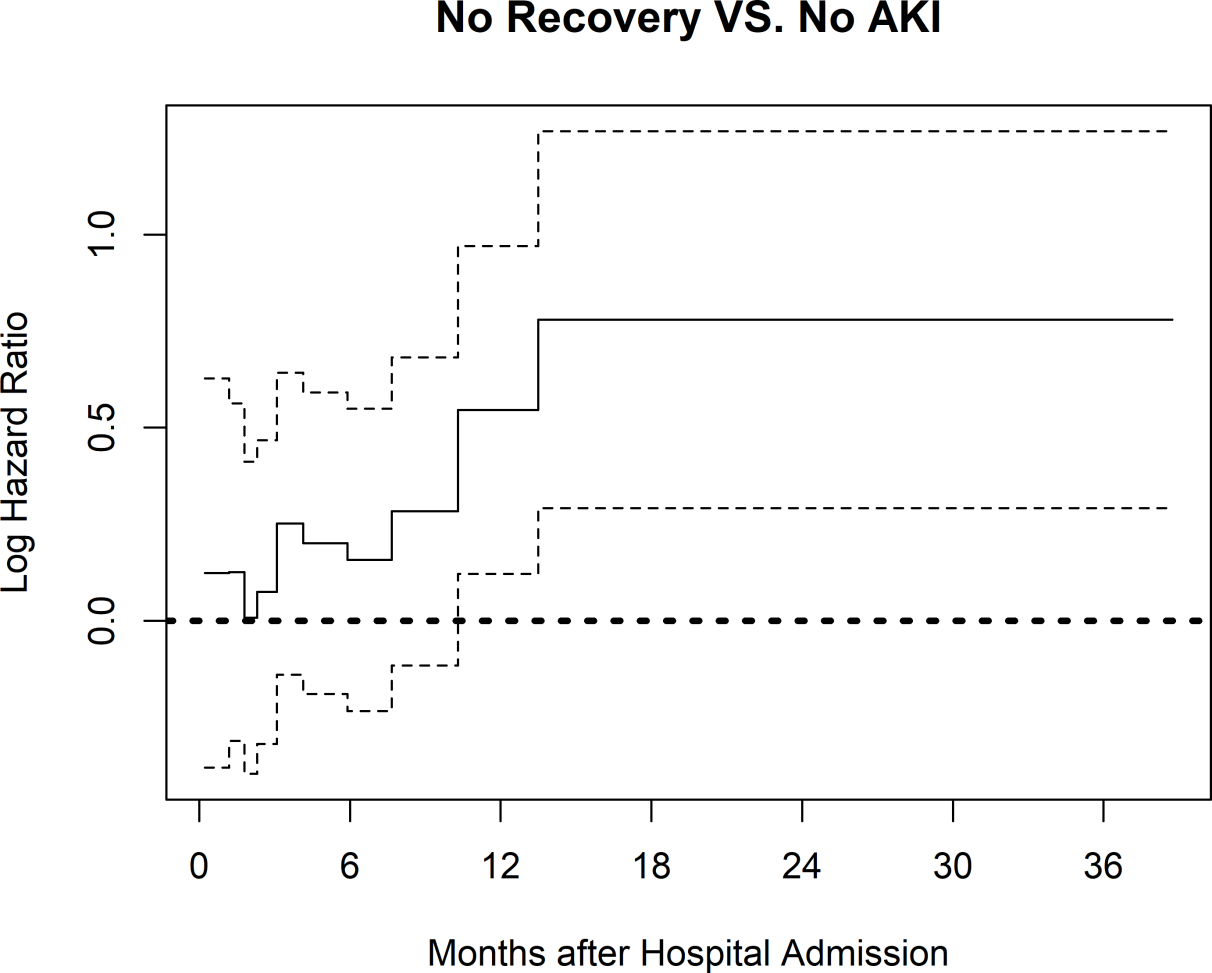
Time-varying covariate effects with 95% confidence intervals of renal recovery at hospital discharge in Gray’s model. Hazard ratios (HR) of Non-recovery vs No AKI from the Gray’s model are reported as the minimum to maximum covariate effect during 10 time intervals. AKI: Acute kidney injury; PSI: Pneumonia Severity Index (on day 1, without age).

Kaplan-Meier (KM) curves showed that non-recovery at hospital discharge was associated with lower survival compared to no AKI (p<0.001). Recovery was associated with similar survival compared to no AKI (p = 0.2) over a maximum follow-up time of 3 years (**Fig 2**). In Gray’s model, non-recovery at hospital discharge was associated with increased mortality compared to no AKI (HR 1.05–2.46, p = 0.01) (**Fig 3**). Patients with recovery had similar survival compared to patients with no AKI (HR 1.01, 95% Confidence Interval [CI] 0.69–1.47, p = 0.96). Other factors associated with increased mortality risk included age (HR 1.58, 95%CI 1.47–1.71, p<0.001), Charlson comorbidity index (HR 1.08, 95%CI 1.04–1.12, p<0.001), male sex (HR 1.30, 95%CI 1.07–1.57, p = 0.007) and day 1 PSI without age (HR 1.01, 95%CI 1.01–1.02, p<0.001) (**Table 3**).

**Table 3:** Factors associated with increased 3-year mortality (n = 1742).

Covariate	Hazard Ratio	95%CI	p-value	Non-proportionality p-value
Age	1.58	1.47–1.71	<0.0001	NA
Male	1.30	1.07–1.57	0.0074	NA
CCI	1.08	1.04–1.12	<0.001	NA
Day1 PSI (no age)	1.01	1.01–1.02	<0.0001	NA
Renal Recovery				
No AKI	1 (reference)			
Recovery vs. No AKI	1.01	0.69–1.47	0.96	NA
Non-recovery vs. No AKI	1.05–2.46*		0.03^+^	0.11

Multivariable Gray’s model was used–Increased hazard ratios imply worse survival.

CCI: Charlson comorbidity index; AKI: Acute kidney injury

PSI: Pneumonia Severity Index

Age: 10 years unit

* For each variable, min and max hazard ratio among 10 time intervals are shown

+ Overall p-value of each variable over all time intervals

### Recovery of renal function at hospital discharge

Higher baseline SCr was strongly associated with *increased* odds of recovery of renal function after AKI (OR 9.16, 95%CI 1.61–52.17, p = 0.01). Patients who had AKI present by day 1 were less likely to recover compared to those who developed AKI after day 1 (OR 0.17, 95%CI 0.09–0.35, p<0.001). Patients receiving RRT during hospitalization had much lower odds of recovery compared to patients who did not (OR 0.08, 95%CI 0.01–0.78, p = 0.03). Moreover, *higher* APACHE III score on day 1 was associated with recovery after AKI (OR 1.03, 95%CI 1.01–1.06, p = 0.008) (**Table 4**). Patients with baseline cardiac disease were less likely to recover (OR 0.53, 95%CI 0.28–1.04) but this did not reach statistical significance (p = 0.06). Other factors including age, gender, race, severity of AKI, Charlson comorbidity index (CCI) and PSI were not significantly associated with recovery after AKI in the model. These results are also consistent after imputing missing data of baseline cardiac disease and AKI on day 1 (**S1 Table**). A model including baseline SCr, AKI on day 1, use of in-hospital RRT Apache III score and CKD showed an area under the ROC curve (AUC) of 0.79 for prediction of recovery by hospital discharge (Hosmer-Lemeshow test statistic: 5.89; p-value 0.66).

**Table 4:** Factors associated with recovery of renal function after AKI in survivors to hospital discharge (n = 262^+^).

	Odds ratio (OR)	95% Confidence interval	p-value
Baseline SCr	9.16	1.61–52.17	0.01
Cardiac disease	0.53	0.28–1.04	0.06
APACHE III score (day 1)	1.03	1.01–1.06	0.008
AKI on day 1	0.17	0.09–0.35	<0.001
In-hospital RRT	0.08	0.01–0.78	0.03

Adjusted for presence of chronic kidney disease at baseline

^+^: missing n = 51 for variable Cardiac Disease and AKI on day 1. The analytic sample size is n = 211.

AKI: acute kidney injury; SCr: serum creatinine; APACHE III: acute physiologic and chronic health evaluation III; RRT: renal replacement therapy

In sensitivity analysis (including non-survivors to hospital discharge), baseline SCr, AKI on day 1 and use in-hospital RRT still remained predictors of recovery after AKI episodes; Moreover, in this model, younger patients were more likely to have recovery compared to older patients (OR 0.98, 95%CI 0.96–1.00, p = 0.02). (**S2 Table**).

## Discussion

In a large cohort of patients hospitalized with community-acquired pneumonia, we found that patients who developed moderate to severe AKI and recovered renal function by hospital discharge had comparable 3-year survival to those patients who never developed AKI. Non-recovery of renal function was associated with more than a 50% increase in the hazard ratio for decreased survival over 3 years.

We recently reported renal recovery in a cohort of 1,243 patients enrolled in the Protocolized Care for Early Septic Shock (ProCESS) trial. Survival in this cohort was similar at 1 year for patients with recovery (definition equivalent to this report) when compared to patients who did not develop AKI [9]. Collectively, findings from ProCESS and GenIMS indicate that recovery of renal function appears to mitigate the well-described hazards of AKI on long-term survival, at least out to 2–3 years. There are several prior studies that examined long-term outcomes after recovery from AKI, often without separation into septic and non-septic AKI. Bucaloiu et al. studied recovery (defined as a return of SCr to 110% of baseline or less by 90 days) in 1610 critically ill patients (with and without sepsis) with no preexisting CKD who developed AKI from any cause. AKI with recovery was still associated with a 48% increase in the risk of death 3.4 years after an episode of AKI, when compared to 3652 propensity-matched controls [19]. This association was no longer present after adjustment for incident CKD, indicating that only patients who subsequently developed CKD were at increased risk of death. Similarly, a retrospective study of 10,518 patients with no baseline CKD who required admission to a surgical ICU and survived to hospital discharge showed that patients with AKI and recovery had a 20% increase in the hazard ratio for decreased survival, compared to those who never developed AKI after 10 years of follow-up [1]. In contrast, Jones et al did not find an association between all-cause AKI with recovery (within 7 days of hospital discharge) and long-term survival in a retrospective study of 3809 hospitalized patients [20]. Recently, Shum et al showed in a cohort of 3687 Chinese critically ill patients that recovery of renal function at 90 days was more frequent in patients with septic AKI compared with those with non-septic AKI (2.5 vs 6.4%, p<0.001), although no differences in in-hospital and 90-day survival were found between the two groups [21]. While it is possible that these differences in results are related to differences in methodology (i.e. timing and definition used for renal recovery) or in follow-up time, recovery after septic AKI could portend a distinct and better long-term prognosis compared to other causes of AKI due to differences in underlying pathophysiology. Moreover, we recently identified five distinct recovery phenotypes (early sustained reversal, late reversal, relapsing AKI with complete recovery at hospital discharge, relapsing AKI without complete recovery at hospital discharge and never reversal AKI) based on the clinical course over 7 days after AKI diagnosis in 16,968 patients with stage 2–3 AKI [22]. Patients with sepsis were more likely to experience relapses after initial AKI reversal and often failed to recover by hospital discharge.

Non-recovery of renal function after AKI has a well-known association with decreased long-term survival [3, 23],[24]. We found associations between AKI occurring after day 1, no use of RRT during hospitalization, higher APACHE III and higher baseline serum creatinine with recovery of renal function at hospital discharge. The presence of AKI on day 1 is often referred to as “community-acquired AKI” (CA-AKI). In a study of 686 hospitalized patients with AKI, Wonnacott et al. showed that CA-AKI was more common and more likely to be severe compared to hospital-acquired AKI (HA-AKI) [25]. Our results add to these findings and suggest that CA-AKI is less likely to recover at hospital discharge, confirming that CA-AKI may represent a distinct phenotype from HA-AKI. Interestingly, while patients with higher baseline SCr clearly have a higher risk of developing AKI [26], we showed that these patients have greater odds of renal recovery. While it is possible that the timing we chose to ascertain renal recovery confounded this association (more difficult for patients with lower baseline SCr to return to baseline by hospital discharge), this association has also been reported when renal recovery was determined 90 days after the AKI episode [3]. It is possible that patients with lower baseline SCr may have more renal functional reserve, and therefore the same AKI stage will represent a larger loss of functioning nephrons compared to patients starting with a higher SCr. This may explain why patients with lower baseline SCr had lower rates of recovery. We also found that a higher APACHE III score on day 1 was associated with increased odds of recovery after AKI. We speculate that among survivors, higher APACHE III scores may select for patients with more recovery potential—in other words, those surviving with high disease severity have greater organ reserve.

A model including presence of AKI on day 1, need for RRT during hospitalization, Apache III score and baseline SCr showed discrete performance (AUC = 0.79) for prediction of recovery in septic AKI. It is important to note that the clinical variables in this model are largely non-modifiable and differ from previously identified predictors of recovery [17, 27]. One of these studies was the Biological Markers of Recovery for the kidney (BioMaRK) study, an ancillary study to the Acute renal failure Trial Network (ATN) study that examined whether inflammatory and apoptosis biomarkers augment risk prediction of renal recovery (defined as being off dialysis at 60 days) and survival. In this cohort 52% of patients had sepsis. Interestingly, a clinical model consisting of age, mean arterial pressure, mechanical ventilation and bilirubin predicted renal recovery with an AUC of 0.73, with modest improvement to an AUC of 0.76 when plasma IL-8 (drawn at the day of RRT initiation) was added to the model [27]. Additional studies are needed to identify clinically modifiable risk factors for recovery and further explore the ability of novel biomarkers to predict recovery after AKI.

There are limitations to our study. First, follow-up time was relatively short even though it is longer than the usual 1 year. The effects of AKI and recovery on survival and ESRD may well require even longer follow-up time. Second, detailed information on microbiological data and antibiotic treatments are not available in this cohort. Third, AKI duration data was available only for a limited number of patients and we did not look at this important variable in the study. Finally, the etiology of AKI was not adjudicated in this study, and exposures such as drugs or contrast may have been in play in addition to sepsis.

In conclusion, recovery of renal function by hospital discharge is associated with excellent long-term survival in patients with septic AKI. Conversely, non-recovery is associated with a significantly increased mortality. While efforts to prevent AKI should continue, therapies and measures to ensure or enhance renal recovery by discharge might reverse the long-term adverse consequences of sepsis-associated AKI.

## Supporting information

S1 Table Sensitivity analysis for identifying risk factors associated with recovery of renal function by hospital discharge after imputation of missing data for cardiac disease and AKI on day 1 using MICE (Multivariate Imputation by Chained Equation). (PDF)

S2 Table Sensitivity analysis for identifying risk factors associated with recovery of renal function by hospital discharge including non-survivors to hospital discharge (n = 314^+^). (PDF)
