# Supplementary material for: Long-term survival in patients with septic acute kidney injury is strongly influenced by renal recovery
Source: PLoS One. 2018 Jun 5;13(6):e0198269. doi: 10.1371/journal.pone.0198269 (PMC5988328; doi:10.1371/journal.pone.0198269)
Supplement: S1 Table — (PDF) [file pone.0198269.s001.pdf]

Supplementary Table 1. Sensitivity analysis for identifying risk factors associated with recovery of renal function by hospital discharge after imputation of missing data for cardiac disease and AKI on day 1 using MICE (Multivariate Imputation by Chained Equation).

	**Odds ratio (OR)**	**95% Confidence interval**	**p-value**
Baseline SCr	13.24	2.52-69.73	0.002
Cardiac disease	0.54	0.28-1.04	0.07
APACHE III score (day 1)	1.03	1.01-1.05	0.003
AKI on day 1	0.13	0.07-0.26	<0.001
In-hospital RRT	0.05	0.01-0.52	0.01

Adjusted for presence of chronic kidney disease at baseline.
Multi-variable logistic regression is used. Cardiac disease and AKI on day 1 were imputed by MICE (Multivariate Imputation By Chained Equations)

AKI: acute kidney injury; SCr: serum creatinine; APACHE III: acute physiologic and chronic health evaluation III; RRT: renal replacement therapy
