# Supplementary material for: Long-term survival in patients with septic acute kidney injury is strongly influenced by renal recovery
Source: PLoS One. 2018 Jun 5;13(6):e0198269. doi: 10.1371/journal.pone.0198269 (PMC5988328; doi:10.1371/journal.pone.0198269)
Supplement: S2 Table — (PDF) [file pone.0198269.s002.pdf]

Supplementary Table 2. Sensitivity analysis for identifying risk factors associated with recovery of renal function by hospital discharge including non-survivors to hospital discharge (n=314^{+})

	**Odds ratio (OR)**	**95% Confidence interval**	**p-value**
Age	0.98	0.96-1.00	0.02
Baseline SCr	5.93	1.44-24.37	0.01
AKI on day 1	0.30	0.18-0.53	<0.001
In-hospital RRT	0.14	0.02-0.87	0.03

* Adjusted for presence of chronic kidney disease at baseline
Multi-variable logistic regression is used
$^{+}$: missing n=10 for variable AKI on day 1. The analytic sample size is n=304.
AKI: acute kidney injury; SCr: serum creatinine; RRT: renal replacement therapy
